# A longitudinal study examining uptake of new recreation infrastructure by inactive adults

**DOI:** 10.1186/s12966-019-0823-4

**Published:** 2019-08-01

**Authors:** Ben J. Smith, Ruth MacKenzie-Stewart, Fiona J. Newton, Tilahun N. Haregu, Adrian Bauman, Robert J. Donovan, Ajay Mahal, Michael T. Ewing, Joshua D. Newton

**Affiliations:** 1 School of Public Health, Level 6, The Charles Perkins Centre, University of Sydney, Sydney, New South Wales 2006 Australia; 2 School of Public Health and Preventive Medicine, Monash University, 553 St Kilda Rd, Melbourne, Victoria 3004 Australia; 3 Department of Marketing, Monash Business School, Monash University, McMahons Rd, Frankston, Victoria 3199 Australia; 4 Melbourne School of Population and Global Health, University of Melbourne, 333 Exhibition St, Melbourne, Victoria 3004 Australia; 5 School of Human Sciences, University of Western Australia, 35 Stirling Highway, Perth, Western Australia 6009 Australia; 6 Nossal Institute for Global Health, University of Melbourne, 333 Exhibition St, Melbourne, Victoria 3004 Australia; 7 Faculty of Business and Law, Deakin University, 221 Burwood Hwy, Burwood, Victoria 3125 Australia

**Keywords:** Physical activity, Recreation facility, Built environment, Cohort study

## Abstract

**Background:**

The built environment is reported to influence physical activity in populations, but longitudinal evidence about the impact of building new physical activity infrastructure is limited. This study aimed to prospectively investigate the uptake and usage of the newly established Peninsula Aquatic and Recreation Centre (PARC), a large multi-purpose recreation facility in Melbourne, Australia.

**Methods:**

Physically inactive adults (*n* = 549) from the City of Frankston were recruited before the opening of PARC and followed up 12 months later to measure frequency of attendance at the Centre, and the purposes and barriers to use. Multivariable methods were used to identity the demographic, cognitive and social predictors of attendance, and the relationship between PARC use and improvements in leisure-time physical activity.

**Results:**

Over 12 months 8.7% of the sampled residents used PARC once per month or more, 17.5% attended less than once per month, and 73.8% did not use the Centre. Lap swimming was the dominant purpose for attendance, and the major barriers were cost of transport and cost of entry. Independent predictors of usage were being female, having children, living within 5 km of the Centre, and expressing strong intentions for use prior to its opening. Use of PARC was not associated with progression to a higher level of total leisure-time physical activity.

**Conclusions:**

While installation of multi-purpose aquatic and recreation facilities may be considered an investment towards physical activity in populations, regular use by inactive people is likely to be low. Strategies to reduce barriers, including cost and transport, and to motivate use should be trialled in order to improve the public health impacts of this form of infrastructure.

## Background

The public health threat presented by physical inactivity has driven research to better understand the determinants of this behaviour and the mix of policies and programs that are likely to increase levels of activity in different population segments [1]. There has been attention to the built environment as an important enabler of physical activity, because of its broad reach, durability, and potential to support social marketing and other interventions [2]. International organisations and public health advocates who have developed action plans to address physical inactivity have placed environmental improvement high on the agenda, calling for governments to formulate policies to bring this about [3].

Although the relationship between the built environment and physical activity is well established, there is yet limited consensus on the environmental elements and characteristics which act as determinants of physical activity in populations. Environmental elements reported to be associated with physical activity include the presence of sidewalks, trails, parks, sports facilities, outdoor exercise equipment, and public transport [4, 5], while attributes of the environment reported to be beneficial encompass aesthetic qualities, path and street connectivity, accessible destinations, safety, and lack of traffic [6, 7]. Those who have reviewed this evidence have noted the dominance of cross-sectional designs that are unable to show causal relationships, the diverse types of physical activity that have been investigated (e.g., walking, recreation, commuting), and the variety of measurement methods used [8].

The availability of recreation infrastructure has been identified in both cross-sectional and longitudinal studies to be an element of the built environment that is positively associated with physical activity [9, 10, 11]. This infrastructure takes a variety of forms, including public parks, sports fields, pay-for-use gymnasiums, swimming pools, and others. A review which examined the relationship between physical activity and different types of recreational infrastructure [12] found stronger associations for outdoor (e.g., parks, trails) than indoor settings (e.g., sports facilities, gymnasiums), and highlighted that parks and trails have been the most studied elements of the recreational environment.

Ecological models posit that the presence of recreational infrastructure may promote physical activity directly, by acting as a cue and opportunity for activity, or indirectly by influencing important intrapersonal determinants (e.g., self-efficacy, social norms) [13, 14]. The Environment Research Framework for Weight Gain Prevention [15] adopts a dual process view to explain these potentially complex relationships, recognising that pathways of direct and indirect influence vary for different aspects of the environment (e.g., parks, pay-for-use gyms) and may be moderated by personal and behaviour related characteristics. Studies examining interactions between characteristics of the built environment and intrapersonal determinants of physical activity have found that the availability of recreational facilities may augment some positive influences, such as intentions, [16, 17] and override negative influences, such as low self-efficacy and fewer perceived benefits [18, 19].

Multi-purpose leisure and aquatic centres are a type of recreational infrastructure established by municipal authorities in many countries, yet they have been given little attention in research concerning the environmental determinants of physical activity. These often provide indoor and/or outdoor swimming pools, gymnasiums and sporting facilities and require significant financial expenditure. Among the few studies examining the impacts of leisure and aquatic facilities are user surveys, which have found health and fitness, relaxation, socialising with friends and family, and self-esteem to be the benefits that are most often reported [20, 21]. From a public health perspective a fundamental question that has not yet been examined is whether the installation of facilities of this type attracts inactive people within communities and contributes to tackling the high prevalence of physical inactivity that is widely reported. In relation to building an ecological understanding of physical activity, a further question is whether local facilities of this nature are used more by community members with certain demographic characteristics and/or cognitive dispositions. The aim of the study reported here was to measure the usage of a newly established leisure and aquatic centre by a cross-sectional sample of physically inactive people, to identify the intrapersonal and social factors that predicted attendance, and to examine whether use resulted in increased levels of leisure-time physical activity.

## Methods

### Peninsula aquatic and recreation Centre (PARC)

PARC is located in the City of Frankston, a local government area (LGA) in southeast Melbourne, Australia, with a population of approximately 134,000. The Centre is positioned adjacent to a railway station, bus routes and the central business district. It offers a 50 m indoor lap pool, learn to swim pools, warm water pool and an aquatic playground area. The Centre is also equipped with a large gymnasium, group exercise rooms, spa, sauna and a wellness centre offering massage and other therapies (https://parcfrankston.com.au/). Onsite parking is available and is free to PARC users for the first 2 h. The establishment cost of PARC was AU$49.7 m and it was opened to the public in September of 2014. Prior to its opening the Centre was promoted extensively in newspapers, letters to residents and public billboards. During its first year of operation casual entry prices to the Centre were AU$8.20 for adults (AU$6.80 off peak), AU$6.50 for children/pensioners ($5.40 off peak), and AU$23.50 for groups of two adults and two children (AU$19.50 off peak).

### Study design

This study investigated attendance at PARC using a cohort design. This cohort was the control arm of the larger MOVE Frankston trial, in which two other experimental groups were exposed to interventions to promote PARC. The methods of this trial have been described previously [22]. The study was conducted with ethics approval from the Monash University Human Research Ethics Committee (Project IDs: CF14/1148–2014000497 and CF14/2059–2014001074).

### Participants and recruitment

Eligible study participants resided in the City of Frankston, were aged 18–70 years of age, participated in 30 min of moderate or more vigorous physical activity on less than 5 days per week, and attended a leisure and aquatic centre or gymnasium less than 3 days per week. Participants were excluded if they were unable to walk independently, had poor English proficiency, could not provide telephone and postal contact information, or had pre-purchased a membership of PARC. The sample size target for the cohort was determined adopting the assumptions that 10% of participants would become regular (weekly) users of PARC and that loss to follow-up would be 20% per year. Therefore, with an alpha of 0.05, and power 0.8, a sample of 500 would provide confidence intervals of +/− 3.5% on prevalence measures in the cohort and an ability to detect 10% difference in outcomes between this group and the intervention arms of the study.

Recruitment was undertaken in August and September of 2014 by telephone calls to a random sample of people with a landline or mobile phone number listed in the Electronic White Pages directory for the Frankston City Council area. Up to six call attempts were made to contact the listed number, and when the person was reached they were asked to state the members of their household in the eligible age range from oldest to youngest. One person within this range was then randomly selected using computer generated random ordering to undertake the screening questions. Individuals meeting the screening criteria were read an explanatory statement about the study and the ethical conditions under which it was being conducted, and those providing verbal consent were administered the baseline survey. If a selected person was not eligible, permission was sought to screen another member of the household.

### Measures

Data were conducted by Computer Assisted Telephone Interview, with the survey being approximately 20 min in duration. The baseline measures were collected in the 6 weeks prior to the opening of PARC, and follow-up measurement was conducted 12 months later.

Physical activity measures were drawn from the Exercise Recreation and Sport Survey, which has been used extensively in Australia to assess participation in organised and non-organised leisure-time activities [23]. This survey asks respondents to identify the exercise, recreation and sports activities undertaken in the past 12 months, and to report the number and duration of their sessions of each of these in the past 2 weeks. Frequency of attendance at PARC was measured by two questions, with the first asking if participants had used the facility at all in the past 12 months, and the second (for users) asking if this attendance was less than once per month, one or two times per month, one or two times per week, or three or more times per week. Those who reported using PARC were asked whether they did so for any of the following purpose(s): lap swimming; use of the warm water wellbeing pool; learn to swim classes; use of the gym; attending an exercise class; undertaking prescribed exercise therapy; or visiting the day spa. Barriers to the use of PARC were measured by asking all participants to rate on a five-point Likert scale (from strongly agree to strongly disagree) the extent to which each of the following prevented their attendance: Centre opening hours; cost of entry; lack of transport; lack of parking; the cost of parking; the Centre not having their preferred exercise facilities; dislike of the social atmosphere at the Centre; and dislike of the physical environment at the Centre.

Single-item measures were used to measure cognitive determinants of physical activity. Respondents were asked to report their agreement, on a five-point Likert scale, with statements about whether they: intended to exercise regularly (intention); [24] considered exercise to be pleasant (attitude); [25] had made a plan about how to exercise regularly (action planning); [26] had the ability to exercise regularly if they wished to (self-efficacy); [27] believed that those who are important to them would approve if they exercised regularly (subjective norm); [25] and, would regret it if they did not exercise regularly (anticipated regret) [28]. The six-item Friendship scale [29] was used to measure social relationships, while a short version of the Functional Comorbidity Index [30] was used to measure existing chronic conditions (e.g., diabetes, arthritis).

Demographic details collected were age, sex, residential address, household composition, educational attainment, occupation, household income, and language spoken at home. In order to determine residential proximity to PARC, the address given by participants was entered into Google Maps so that the driving distance to the Centre could be generated.

### Statistical analysis

The ordinal measure of frequency of PARC attendance was recoded to categorise usage in the previous 12 months as none, occasional (less than once per month) and regular (one or two times per month, or more). Descriptive proportions were calculated for levels of PARC use, the reasons for use, and perceived barriers to attendance. Analysis of baseline predictors of PARC use over 12 months was undertaken by stratifying the levels of use by the demographic, health status, cognitive and social characteristics of participants. In this analysis residential proximity to PARC was categorised as less than 5 km, or 5 km or more. The other cognitive variables (intention, attitude, action planning, self-efficacy, subjective norm, anticipated regret) that were measured on Likert scales were dichotomised (agree vs neutral/disagree), to differentiate those displaying the cognitive characteristic from those who did not.

Because levels of regular attendance at PARC were low, those who were regular or occasional users were combined into a single group (labelled ‘users’). Crude odds ratios were calculated to identify the demographic, health status, cognitive and social characteristics that had bivariate associations with PARC attendance. Stepwise multivariable logistic regression was undertaken to identify the factors that independently predicted PARC attendance at 12 months, with a *p* < 0.1 threshold applied for inclusion of variables at each step in modelling.

In order to examine whether attendance at PARC was independently related to increased leisure-time physical activity, total minutes of moderate- and vigorous-intensity activity reported in the past 2 weeks were calculated at both baseline and 12 months. The physical activity level of participants at each time point was classified as sedentary (less than 30 min per week), low active (30–149 min per week), or sufficiently active (150 min or more per week). PARC use and baseline level of physical activity were entered into a stepwise multivariable logistic regression model, together with the demographic, health status, cognitive and social variables, to identify factors associated with progression to a higher level of physical activity over 12 months (i.e., sedentary or low active, to a higher level). A *p* < 0.1 threshold was applied for inclusion of variables at each step in modelling.

## Results

During telephone recruitment 61% of contacted individuals were found to meet the study eligibility criteria. Of these 55% were enrolled, 549 of whom were allocated to the control arm of the MOVE-Frankston trial, which was the cohort followed for this study.

As Table 1 shows, the majority of participants were female and most were in the 35–54 years or 55–70 years age groups. Three-quarters lived in a household with two or more adults, and just over one-third had one or more children under 18 years of age living with them. Most participants had attained a post-school qualification, with a vocational qualification reported more often than a university degree. The majority were in paid employment, which was mostly full-time. Although around 10% of participants did not disclose their household income, there was good representation across the lower, middle, and upper brackets. About three-in-five participants resided five kilometres or more from PARC.

**Table 1:** Characteristics of study participants

	Baseline (*N* = 549)		12 months (*N* = 389)	
	N	%	N	%
Gender^a^				
Male	205	37.3	148	38.0
Female	317	57.7	227	58.4
Age^a^				
18–34 years	57	10.4	38	9.8
35–54 years	216	39.3	145	37.3
≥ 55 years	237	43.2	181	46.5
Adults in household				
One	133	24.2	95	24.4
Two or more adults	416	75.7	294	75.6
Children in household				
None	350	63.8	249	64.0
One or more	199	36.2	140	36.0
Education^a^				
High school or less	175	31.9	115	29.6
Vocational qualification	222	40.4	160	41.1
University degree	140	25.5	106	27.2
Employment status^a^				
Employed (F/T)	238	43.4	150	38.6†
Employed (P/T)	118	21.5	90	23.1
Others	166	30.2	135	34.7
Household income^a^				
≤ AU$39,999	148	27.0	111	28.5
AU$40,000 - 79,999	185	33.7	131	33.7
≥ AU$80,000	171	31.1	115	29.6
Main Language^a^				
English	508	92.5	368	94.6
Other	14	2.6	7	1.8
Distance from PARC^a^				
< 5 kms	211	38.4	145	37.3
≥ 5 kms	316	57.6	228	58.6

^a^data missing for participants; † difference with those lost to follow-up (*p* < 0.01)

The 12-month follow-up measures were completed by 70.9% of those recruited. Study participants successfully followed up were significantly less likely than those lost to follow-up to be in full-time employment, but did not differ in any other demographic characteristic.

**Fig. 1:**
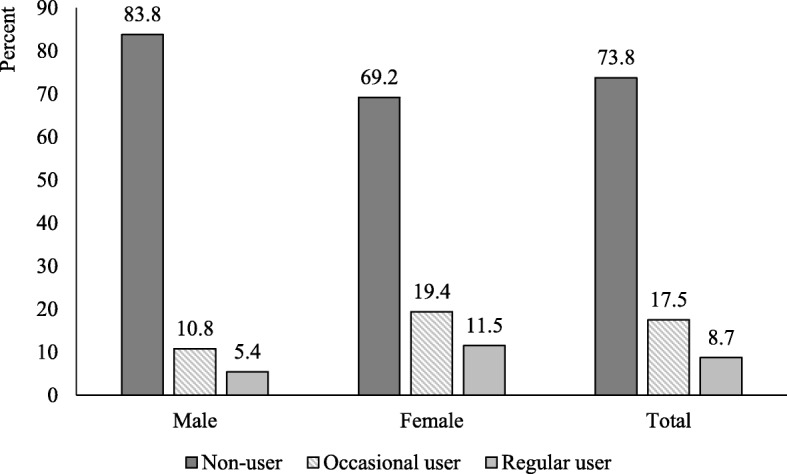
Attendance at PARC over 12 months by gender for physically inactive residents of Frankston City

As shown in Fig. 1, at the 12-month follow-up 73.8% of study participants were classified as non-users of PARC, 17.5% as occasional users (less than once per month), and 8.7% as regular users (once per month or more). Among the regular users just over half (4.6% of the total sample) reported attending PARC at least once per week.

### Reasons and barriers for PARC use

The most common reasons given for using PARC were lap swimming (70.4%), followed by using the warm-water pool (38.8%). Markedly lower proportions reported using the gymnasium (7.2%), undertaking group exercise classes (6.2%), or attending swimming classes (6.2%).

The most reported barriers to PARC use were the cost of parking (39.0%), cost of entry to the Centre (32.3%) and lack of parking (20.3%). It was notable that each of these barriers was reported significantly more often by occasional than regular users; 55.2% vs 20.6% for cost of parking, 43.3% for 29.4% for cost of entry, and 29.4% vs 14.7% for lack of parking, respectively. Lack of parking was more often reported by women than men (23.7% vs 14.4%), and cost of entry more often by those in the lowest category of income (42.7%) than people in the middle (27.7%) and upper categories (25.5%). Less common barriers were limited facilities at PARC (7.8%), the social climate at the Centre (7.4%), the physical environment at the Centre (7.3%), lack of transport (4.7%) and unsuitable opening hours (3.0%).

### Predictors of PARC usage and leisure time physical activity

Table 2 shows the proportions of participants from different demographic and health status sub-groups who were classified as non-users, occasional users or regular users of PARC over the preceding 12 months. Univariable logistic regression revealed that those most likely to make any use of PARC were females (odds ratio (OR) 2.30, 95% confidence interval (CI), 1.37–3.87), people in a household with one or more children (OR 2.94, 95% CI 1.85–4.69), and those employed on a part-time basis (OR 2.79, 95% CI 1.56–4.99). In addition, those with a high school education had a higher likelihood of PARC attendance than those with a vocational qualification (OR 0.49, 95% CI 0.28–0.84), people from low income compared with middle income households (OR 0.52, 95% CI 0.29–0.93), and those without chronic conditions compared with those reporting two or more (OR 0.39 95% CI 0.22–0.69). Those residing within 5 km of PARC were more likely to have used the centre than those who lived ≥5 km away (OR 0.58, 95% CI 0.37–0.93).

**Table 2:** Usage of PARC at 12 months by socio-demographic characteristics of residents: univariable regression

Socio-demographic characteristics	Non-user		Occasional user		Regular user		PARC use
	N	%	N	%	N	%	Crude OR (95% CI)
Gender							
Male	124	83.8	16	10.8	8	5.4	1.00
Female	157	69.2	44	19.4	26	11.5	2.30 (1.37, 3.87)
Age							
18–34 yrs	30	86.0	4	7.0	4	7.0	1.00
35–54 yrs	92	75.5	39	18.1	14	6.5	2.16 (0.92, 5.05)
≥ 55 yrs	151	87.3	14	5.9	16	6.8	0.75 (0.31, 1.78)
Children in household							
None	203	81.5	28	11.2	18	7.2	1.00
One or more	84	60.0	40	28.6	16	11.4	2.94 (1.85, 4.69)
Education							
High school or less	76	66.1	27	23.5	12	10.4	1.00
Vocational qualification	128	80.0	21	13.1	11	6.9	0.49 (0.28, 0.84)
University degree	75	70.8	20	18.9	11	10.4	0.81 (0.46, 1.42)
Employment							
Employed (F/T)	120	80.0	18	12.0	12	8.0	1.00
Employed (P/T)	53	58.9	24	26.7	13	14.4	2.79 (1.56, 4.99)
Others	108	80.0	18	13.3	9	6.7	1.00 (0.56, 1.79)
Household income							
≤ AU$39,999	75	67.6	27	24.3	9	8.1	1.00
AU$40,000 -79,999	105	80.2	14	10.7	12	9.2	0.52 (0.29, 0.93)
≥ AU$80,000	81	70.4	23	20.0	11	9.6	0.87 (0.50, 1.54)
Chronic conditions							
None	57	63.3	23	25.6	10	11.1	1.00
One	71	68.9	24	23.3	8	7.8	0.79 (0.43, 1.42)
Two or more	154	81.5	20	10.6	15	7.9	0.39 (0.22, 0.69)
Distance from PARC							
< 5kms	97	66.9	29	20.0	19	13.1	1.00
≥ 5 kms	177	77.6	37	16.2	14	6.1	0.58 (0.37, 0.93)

PARC usage at 12 months by study participants having various cognitive and social characteristics is shown in Table 3. Those who expressed intentions to attend PARC at baseline showed a higher prevalence of using the Centre, but the odds ratio for this association was marginally non-significant (OR 2.08, 95% CI 0.98–4.42). There was little variation in PARC usage across sub-groups of the cognitive determinants of physical activity, including attitudes, subjective norm, action planning, self-efficacy, and anticipated regret. Level of personal friendship and support was not related to PARC usage.

**Table 3:** Usage of PARC at 12 months by cognitive and social characteristics of residents: univariable regression

Cognitive and social characteristics	Non-users		Occasional users		Regular users		PARC use
	N	%	N	%	N	%	Crude OR (95% CI)
Intention							
Neutral/ negative	48	84.2	8	14.0	1	1.8	
Positive	238	71.9	60	18.1	33	10.0	2.08 (0.98–4.42)
Attitude							
Neutral/ negative	59	77.6	13	17.1	4	5.3	
Positive	227	72.8	55	17.6	30	9.6	1.30 (0.72–2.35)
Subjective norm							
Neutral/ negative	10	71.4	3	21.4	1	7.1	
Positive	276	73.8	65	17.4	33	8.8	0.89 (0.27–2.90)
Action planning							
Neutral/ negative	134	76.1	30	17.1	12	6.8	
Positive	152	71.7	38	17.9	22	10.4	1.30 (0.81–2.08)
Self-efficacy							
Neutral/ negative	23	69.7	7	21.2	3	9.1	
Positive	262	74.0	61	17.2	31	8.8	0.81 (0.37–1.76)
Anticipated regret							
Neutral/ negative	32	78.1	9	22.0	0	0	
Positive	254	73.2	59	17.0	34	9.8	1.30 (0.60–2.83)
Friendship							
Median (IQR)	28	(26,30)	28.5	(27, 30)	27	(24, 30)	1.00 (0.94–1.07)

Table 4 shows the factors identified by multivariable modelling as significant predictors of PARC attendance. These were: being female (OR 2.85, 95% CI 1.56–5.22); having one or more children in the household (OR 3.35, 95% CI 1.89–5.98); and expressing intentions to use the Centre at baseline (OR 2.52, 95% CI 1.07–5.96). In addition, people living less than 5 km from PARC were more like to be users relative to those residing 5 km or more away (OR 0.50, 95% CI 0.28–0.89).

**Table 4:** Predictors of PARC attendance over 12 months: multivariable regression

Predictor	PARC use
	Adjusted OR (95 CI)
Gender	
Male	1.00
Female	2.85 (1.56–5.22)
Children in household	
None	1.00
One or more	3.35 (1.89–5.98)
Distance from PARC	
< 5kms	1.00
≥ 5kms	0.50 (0.28–0.89)
Intention	
Neutral/ negative	1.00
Positive	2.52 (1.07–5.96)

Further stepwise modelling found that PARC users were not significantly more likely than non-users to show improvements in their level of leisure-time physical activity over 12 months. Having intentions to exercise regularly at baseline was the strongest predictor of improvement in leisure-time physical activity at follow-up.

## Discussion

Establishment of indoor aquatic and leisure centres requires substantial planning and investment of public funds, yet this is the first longitudinal study we are aware of to investigate use of a new facility of this type by physically inactive adults in a local community. Over 12 months about one-tenth of those classified as physically inactive visited PARC a few times, and less than one in 20 were weekly users. The findings that users of PARC mostly attended on an occasional basis, and that use was not associated with progression to a higher level of total leisure-time physical activity, indicates that the introduction of the Centre is likely to have made only a small contribution towards increasing physical activity in the population.

Analysis of the predictors of attendance at PARC revealed that women and those with children are likely to benefit most from the provision of this type of facility. This greater usage is consistent with population data concerning organised physical activity in Australia which show that a higher proportion of women than men undertake activity in fitness, leisure or indoor sports centres [31]. The greater levels of attendance by those with one or more children indicates that indoor, multi-purpose centres such as PARC could be an attractive way of engaging children in active recreation.

The finding that those living in closest proximity to PARC (< 5 km) were more likely to be attenders shows that ease of access influences the usage of recreational infrastructure, even when this is centrally located and offers a range of high quality facilities that might be attractive to people in the wider region. In support of this, a survey of users at four aquatic and leisure centres in Victoria, Australia, found that being close to home was a major reason for selecting their particular facility [32]. Longitudinal studies investigating the impact of developments to parks [33], greenways [34], and cycle paths [35] have also found that the highest users were people living in the nearest proximity. It is possible that the advertising of new facilities such as PARC as sites where residents can undertake physical activity may increase perceived barriers to activity for those who do not live close to these opportunities. The challenge that this presents for policy makers is that facilities such as PARC have high establishment costs and are not feasible to replicate in multiple locations in communities. Addressing transport access is therefore critical, and at PARC this entailed selection of a site on major bus and rail routes and provision of a large parking lot with 2 h of free parking for Centre users. Despite these initiatives, cost of parking and availability of parking were two of the most reported barriers to use of the Centre, which may have been a perception arising from negative publicity about parking fees and fines in the Frankston CBD. This highlights the importance of including information about ease of transport access in promotion of recreational facilities.

With respect to economic factors that may influence the use of PARC, the multi-variable model found that level of household income did not predict attendance. On the other hand, cost of parking and cost of entry were two out of the three most reported barriers to using the Centre. An analysis of predictors of adherence to leisure centre programs in a deprived area of London found that ‘lack of money’ was a barrier reported four times more often by those who dropped out than those who continued [36]. Qualitative studies have also reported that cost is a barrier to participation in various types of organised and centre-based physical activity for low income people [37]. Fee setting is a complex issue for managers of aquatic and recreation facilities, who are expected to develop a sustainable business model for their operations. At PARC entry fees are discounted for pension beneficiaries, families, and those attending in off-peak times, and further reductions are likely to require a higher level of government subsidisation of the Centre.

The only cognitive predictor of PARC attendance was intentions to use the Centre. This is consistent with a review of research concerning interactions between physical activity cognitions and the built environment, which found that intentions consistently had a positive impact on behaviours when recreational facilities were accessible and convenient [16]. It was notable that self-efficacy, a well-established determinant of physical activity [1], was not associated with Centre usage. This may be because the dependent variable in this analysis was any level of PARC use, rather than regular participation. It is also possible that the use of single item measures of the cognitive variables may have provided insufficient sensitivity to discriminate between participants and detect associations with PARC use. The findings concerning the predictive role of intentions raise questions about what the intrinsic and/or extrinsic motivators were for use of PARC [38]. Further research to examine why some inactive people formed these intentions, while others did not, could inform promotional strategies for facilities of this type.

The finding that the new PARC facility attracted limited numbers of inactive people on a regular basis indicates that complementary strategies to promote usage are needed. Social marketing methods have been used successfully in programs to increase physical activity [39] and are well suited to promoting the use of aquatic and leisure facilities. Innovative strategies, including the Leisure Card scheme in the United Kingdom [40] and the *Give it a Go* program in the London borough of Camden [41], have used social marketing techniques to facilitate access to aquatic and leisure centres. Reviews of social marketing strategies to promote physical activity have concluded that these are most effective when a range of benchmark criteria are adopted in intervention development, including formative research, audience segmentation, clarification of the exchange (or behavioural choice) required, and attention to all elements of the marketing mix (product, price, placement and promotion) [42, 43]. An opportunity in the marketing of leisure and aquatic centres is that it is possible to promote a core product (physical activity and its benefits), and an actual product (a facility where this can be done) [43].

A strength of this study was that a random cross-section of inactive residents was recruited into the cohort. While generalisability to the local population cannot be determined, the higher representation of women, people aged 55–70 years, and those without university education is consistent with population data about physically inactive adults in the state of Victoria [44]. A limitation of this study was that the sample size was insufficient to examine interactions between predictors of PARC usage, which would have enabled exploration of the potential moderating effects of factors such as age and household income upon other predictors of attendance. A further limitation was that PARC usage was measured by an ordinal, self-report measure that is vulnerable to recall bias and did not record the actual number of visits by study participants. This method of measurement was used because it was not possible to obtain admission records from PARC that could be linked to individuals enrolled in the study.

## Conclusion

A major impetus for the development of leisure and aquatic facilities in Australia, and in other parts of the world, is to not only improve local recreational opportunities, but to facilitate health enhancing physical activity. The study reported here is the first longitudinal investigation of the uptake of a new facility of this type by inactive adults in a surrounding locality. It found that around three-quarters of residents did not attend the Centre, that those who attended mostly did so on an occasional basis, and that users did not appear to increase their overall level of leisure-time physical activity. Comprehensive strategies to increase utilisation of leisure and aquatic facilities by inactive people are needed, which not only address barriers to attendance but incentives and support that may promote use, so that the potential public health benefits of this form of recreational infrastructure can be more fully realised.

## Data Availability

The datasets used and/or analysed during the current study are available from the corresponding author on reasonable request.

## Abbreviations

CI: Confidence interval
OR: Odds ratio
PARC: Peninsula Aquatic and Recreation Centre
